# Impact of remnant cholesterol on acute ischemic stroke prognosis: a nationwide cohort analysis stratified by non-alcoholic fatty liver disease status

**DOI:** 10.3389/fneur.2025.1472871

**Published:** 2025-01-30

**Authors:** Sili Jiang, Aoming Jin, Wenli Xing, Jing Jing

**Affiliations:** ^1^Department of Cerebrovascular Diseases, Suining Central Hospital, Suining, Sichuan, China; ^2^Department of Neurology, Beijing Tiantan Hospital, Capital Medical University, Beijing, China; ^3^Tiantan Neuroimaging Center of Excellence, China National Clinical Research Center for Neurological Diseases, Beijing Tiantan Hospital, Capital Medical University, Beijing, China

**Keywords:** remnant cholesterol, ischemic stroke, non-alcoholic fatty liver disease, stroke prognosis, CNSR-III

## Abstract

**Background:**

Remnant cholesterol (RC) is an emerging non-traditional risk factor for cardiovascular diseases that has garnered increasing attention. In addition, non-alcoholic fatty liver disease (NAFLD) may interact synergistically with RC. This study aimed to evaluate the association between RC and functional outcomes in ischemic stroke patients and to investigate the potential interaction effect between RC and NAFLD.

**Methods:**

This study utilized data from the Third China National Stroke Registry (CNSR-III), which includes ischemic stroke patients monitored for 3 months post-stroke onset. RC was calculated by subtracting both low-density lipoprotein cholesterol (LDL-C) and high-density lipoprotein cholesterol (HDL-C) from total cholesterol. Poor functional outcomes were defined as a modified Rankin Scale (mRS) score of 3–6 at the 3-month follow-up. Multivariable logistic regression analyses were conducted to determine the association between RC and functional outcomes. Interaction effect analysis was performed to investigate how NAFLD modifies the relationship between RC and prognosis.

**Results:**

Among the 7, 234 participants, the mean age was 62.96 ± 11.44 years and 4,572 (63.2%) were male individuals. Compared to the lowest quartile of RC (Q1), the highest quartile of the RC (Q4) was associated with a lower risk of poor functional outcomes (OR: 0.98, 95% CI: 0.96–1.00). Meanwhile, we observed a similar relationship between RC and poor functional outcomes in patients with NAFLD (OR: 0.96, 95% CI: 0.93–0.99); however, in those without NAFLD, there was no significant association between RC and poor functional outcomes.

**Conclusion:**

We found an inverse relationship between RC levels and poor functional outcomes in patients with ischemic stroke, which was influenced by NAFLD. Future studies are needed to determine the optimal target levels of RC in NAFLD patients.

## 1 Introduction

Remnant cholesterol (RC) refers to the cholesterol content of all triglyceride-rich lipoproteins, including very low-density lipoprotein (VLDL), synthesized by the liver; chylomicrons, derived from the intestinal tract; and intermediate-density lipoproteins (1). RC is increasingly recognized as a key factor in residual cardiovascular risk (2–4), helping to explain cardiovascular risks and mortality that persist even after achieving target low-density lipoprotein cholesterol (LDL-C) levels (5, 6). Dyslipidemia is not only strongly linked to atherosclerosis and stroke (7, 8) but also affects the smooth muscle and endothelial function of cerebral arteries (9). Research has also highlighted the pro-inflammatory and anti-inflammatory effects of different lipid components (10). In addition, lipids have been found to correlate with brain-derived neurotrophic factor (BDNF), which is essential for the development, maintenance, and recovery of the central nervous system (11, 12). These findings suggest a potential link between RC and stroke outcomes. However, the impact of RC on ischemic stroke outcomes remains unclear.

Non-alcoholic fatty liver disease (NAFLD) is the most common chronic liver disease, affecting approximately 40% of acute stroke patients (13). Although existing studies suggest that patients with NAFLD may have worse stroke prognoses (14), research on the underlying mechanisms remains limited. In particular, the relationship between NAFLD and lipid metabolism-related factors has not been fully elucidated. Patients with NAFLD showed increased expression levels of ANGPTL3, which regulates residual cholesterol synthesis, leading to elevated remnant cholesterol (15). Conversely, RC levels were positively associated with NAFLD risk (16). In addition, higher serum levels of remnant cholesterol were linked to more severe hepatic steatosis, independent of conventional lipid parameters (17). A previous study found that NAFLD was independently associated with higher circulating remnant cholesterol, and elevated remnant cholesterol levels were shown to predict major adverse cardiovascular and cerebrovascular events in NAFLD patients (18).

In this analysis of the CNSR-III trial, we aimed to examine the association between residual cholesterol and stroke outcomes in a large, prospective, multicenter cohort. Furthermore, we investigated the interaction between residual cholesterol and NAFLD in patients with ischemic stroke.

## 2 Methods

### 2.1 Study design and participants

This analysis utilized data from the Third China National Stroke Registry (CNSR-III), which is a comprehensive, prospective, multicenter cohort registry dedicated to patients with acute ischemic cerebrovascular events (19). The cohort included 15,166 patients from 201 sites across eastern, western, and central China. The inclusion criteria were as follows: (1) individuals aged 18 years or older; (2) individuals diagnosed with ischemic stroke or transient ischemic attack; and (3) individuals enrolled within 7 days of symptom onset. The exclusion criteria were as follows: (1) individuals with transient ischemic attack; (2) individuals with viral hepatitis; (3) individuals with a history of substantial alcohol consumption; (4) individuals lacking essential measurements, such as height, weight, and waist circumference, or critical laboratory data required for NAFLD and residual cholesterol assessment; and (5) individuals with no follow-up modified Rankin Scale (mRS) scores recorded. Ethical approval for CNSR-III was granted by the ethics committees of the participating institutions, and written informed consent was obtained from the patients or their legal representatives.

### 2.2 Baseline data collection

Experienced research coordinators collected baseline data from the enrolled patients through face-to-face interviews or electronic medical records, following a standardized protocol for data collection. The collected data included information regarding patient demographics, body mass index (BMI, calculated as weight in kilograms divided by the square of height in meters), smoking status, alcohol consumption, the National Institutes of Health Stroke Scale (NIHSS) score at admission, and medical history. Medical history included hypertension, diabetes, and coronary artery disease. Ischemic stroke etiology was classified according to the Trial of ORG 10172 in Acute Stroke Treatment (TOAST) criteria. The laboratory parameters, including total cholesterol (TC), triglycerides (TG), low-density lipoprotein cholesterol (LDL-C), and high-density lipoprotein cholesterol (HDL-C), were recorded. In addition, details regarding medications administered during hospitalization and the utilization of reperfusion therapy were documented.

### 2.3 Measurements of the RC

The fasting blood samples collected in EDTA tubes were obtained within 24 h of admission from 171 study sites. The samples were transported to the central laboratory at Beijing Tiantan Hospital under a maintained cold chain and stored at −80°C until analysis. The remnant cholesterol (RC) was calculated as total cholesterol minus the measured low-density lipoprotein cholesterol (LDL-C) and high-density lipoprotein cholesterol (HDL-C) (3).

### 2.4 NAFLD screening index

The fatty liver index (FLI) (20) was calculated using the measurements of triglycerides (mmol/L), *γ*-glutamyl transferase (GGT) levels (U/L), and waist circumference (WC) (cm), with the formula as follows:

$\begin{array}{l} FLI=\left[e\left(\begin{array}{l} 0.953\times \ln\left(\mathrm{triglycerides}\right)+0.139\times BMI+\\ 0.718\times \ln\left(GGT\right)+0.053\times WC-15.745 \end{array}\right)\right]/\\ \left[1+e\left(\begin{array}{l} 0.953\times \ln\left(\mathrm{triglycerides}\right)+0.139\times BMI+\\ 0.718\times \ln\left(GGT\right)+0.053\times WC-15.745 \end{array}\right)\right]\times 100 \end{array}$

where GGT = γ-glutamyl transferase, WC = waist circumference, and BMI = body mass index. Based on previously reported standards for Asian populations, the patients were categorized into three groups: (I) no NAFLD: FLI < 25 for the male participants and FLI < 10 for the female participants; (II) possible NAFLD: 25 ≤ FLI < 35 for the male participants and 10 ≤ FLI < 20 for the female participants; (III) NAFLD: FLI ≥ 35 for the male participants and FLI ≥ 20 for the female participants (21).

### 2.5 Outcomes and follow-up

The participants were followed up with face-to-face interviews at 3 months by trained research coordinators using a standardized interview protocol (19). The poor functional outcomes were defined as a modified Rankin Scale score of 3 to 6 at 3 months (22).

### 2.6 Statistical analysis

All participants were classified into four groups based on the quartiles of the fasting remnant cholesterol levels, and the baseline characteristics were compared across these quartiles. Continuous variables were presented as mean (SD) or median (interquartile range), while categorical variables were expressed as frequency (%). The trend across the quartiles was analyzed using the Cochran–Armitage test for the categorical variables, and ANOVA or the Kruskal–Wallis test was used for the continuous variables, as appropriate.

To identify significant predictors of the poor outcomes at the 3-month follow-up, both univariable and multivariable analyses were performed using logistic regression models to estimate odds ratios (ORs) and 95% confidence intervals (CIs). Multivariable regression models were employed to adjust for potential confounding factors, including age, sex, smoking status, alcohol consumption, BMI, hypertension, diabetes, coronary heart disease (CHD), NIHSS score at admission, TOAST classification, antihypertensive drug use, and use of antiplatelet agents.

A restricted cubic spline (RCS) model was used to explore the potential non-linear relationship between the remnant cholesterol (RC) levels and poor functional outcomes. The RCS method allows for flexible modeling of non-linear associations by fitting cubic spline functions at predefined knots. The knots were placed at the 5th, 50th, and 95th percentiles of the RC levels. The significance of non-linearity was assessed using the likelihood ratio test, comparing the model with linear terms only to the model with non-linear spline terms. The resulting RCS plot was constructed with the RC values on the x-axis and the odds ratios (ORs) with 95% confidence intervals (CIs) on the y-axis to visually depict the association.

Interaction between the remnant cholesterol groups and non-alcoholic fatty liver disease (NAFLD) categories was assessed by including interaction terms in the models. All statistical analyses were conducted using SAS, version 9.4 (SAS Institute). Two-sided tests were employed, and *p*-values less than 0.05 were considered statistically significant.

## 3 Results

### 3.1 Baseline characteristics

**Figure 1:**
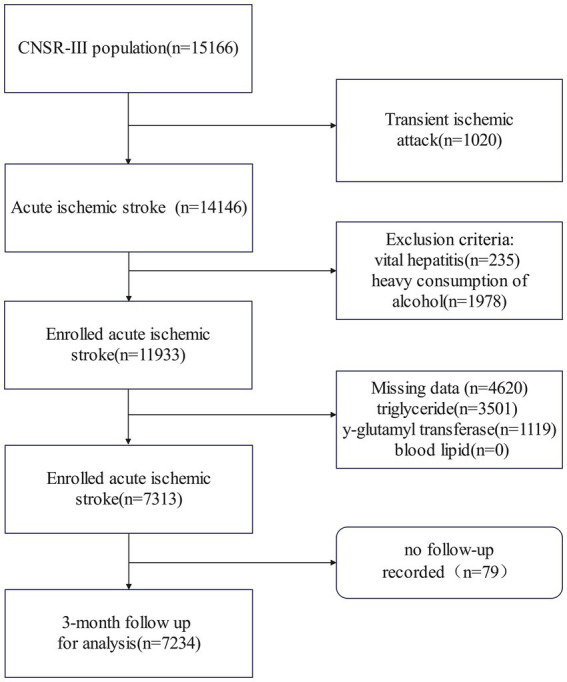
Flowchart of patient selection. CNSR-III stands for the Third China National Stroke Registry.

The Third China National Stroke Registry initially enrolled 15,166 participants. After excluding patients with diagnoses of transient ischemic attack (1,020) or viral hepatitis (235), as well as those with incomplete laboratory or clinical data (4,620), heavy alcohol consumption (1,978), and missing 3-month follow-up Modified Rankin Scale (mRS) records (79), a total of 7,234 patients were included in the final analysis (Figure 1). These patients had a mean age of 63.00 years (SD = 11.44), and the male participants accounted for 63.2% of the cohort. The initial median NIHSS score was 3 (interquartile range: 2–6). The patients with higher remnant cholesterol (RC) levels were more likely to be women, had a higher body mass index, had a history of diabetes and hypertension, had elevated levels of triglycerides and total cholesterol, and had lower levels of high-density lipoproteins (HDL) and low-density lipoproteins (LDL) at admission (Table 1).

**Table 1:** Clinical characteristics of participants stratified by quartiles of remnant cholesterol.

		Quartiles of remnant cholesterol				
	Total	Q1	Q2	Q3	Q4	P for trend
n	7,234	1810	1819	1797	1808	
Age (years); mean (SD)	62.96 (11.44)	65.86 (11.15)	63.79 (11.38)	61.85 (11.29)	60.31 (11.19)	<0.001
No. of male patients (%)	4,572 (63.2)	1,172 (64.8)	1,189 (65.4)	1,135 (63.2)	1,076 (59.5)	0.001
BMI (kg/m^2^); mean (SD)	24.68 (3.34)	23.97 (3.25)	24.53 (3.26)	24.90 (3.41)	25.33 (3.29)	<0.001
No. of current smokers (%)	1772 (24.5)	417 (23.0)	422 (23.2)	485 (27.0)	448 (24.8)	0.02
No. of current drinkers (%)	198 (2.7)	198 (2.7)	70 (3.9)	43 (2.4)	45 (2.5)	0.008
Medical history (%)						
Hypertension	4,604 (63.6)	1,091 (60.3)	1,115 (61.3)	1,174 (65.3)	1,224 (67.7)	<0.001
Previous Stroke	1,693 (23.4)	428 (23.6)	445 (24.5)	421 (23.4)	399 (22.1)	0.392
Previous TIA	143 (2.0)	48 (2.7)	34 (1.9)	32 (1.8)	29 (1.6)	0.111
Diabetes	1785 (24.7)	362 (20.0)	444 (24.4)	422 (23.5)	557 (30.8)	<0.001
CHD	785 (10.9)	229 (12.7)	193 (10.6)	179 (10.0)	184 (10.2)	0.037
PAD	55 (0.8)	16 (0.9)	13 (0.7)	13 (0.7)	13 (0.7)	0.921
Renal insufficiency	63 (0.9)	12 (0.7)	12 (0.7)	15 (0.8)	24 (1.3)	0.101
Baseline NIHSS score; median (IQR)	3.00 [2.00, 6.00]	4.00 [2.00, 7.00]	4.00 [2.00, 6.00]	3.00 [2.00, 6.00]	3.00 [2.00, 6.00]	<0.001
TOAST subtype, no. (%)						<0.001
Large artery at herosclerosis	1843 (25.5)	440 (24.3)	488 (26.8)	462 (25.7)	453 (25.1)	
Cardio-embolism	507 (7.0)	185 (10.2)	136 (7.5)	110 (6.1)	76 (4.2)	
Small artery occlusion	1,562 (21.6)	358 (19.8)	404 (22.2)	379 (21.1)	421 (23.3)	
Another cause	92 (1.3)	20 (1.1)	27 (1.5)	25 (1.4)	20 (1.1)	
Undetermined cause	3,230 (44.7)	807 (44.6)	764 (42.0)	821 (45.7)	838 (46.3)	
NAFLD, no. (%)						<0.001
NAFLD	3,780 (52.3)	552 (30.5)	730 (40.1)	1,039 (57.8)	1,459 (80.7)	
Possible NAFLD	1,350 (18.7)	395 (21.8)	406 (22.3)	350 (19.5)	199 (11.0)	
No NAFLD	2,104 (29.1)	863 (47.7)	683 (37.5)	408 (22.7)	150 (8.3)	
Medication, no. (%)						
Antihypertensive	3,361 (46.8)	754 (42.0)	848 (47.0)	859 (48.3)	900 (50.1)	<0.001
Antiplatelet	6,939 (96.7)	1723 (95.9)	1732 (96.0)	1734 (97.6)	1750 (97.4)	0.003
Lipid lowering	6,948 (96.8)	1738 (96.7)	1741 (96.5)	1721 (96.8)	1748 (97.3)	0.544
Statins	6,934 (96.7)	1736 (96.6)	1738 (96.3)	1720 (96.8)	1740 (96.9)	0.813
Fibrates	24 (0.3)	3 (0.2)	5 (0.3)	6 (0.3)	10 (0.6)	0.226
Intravenous alteplase	672 (9.3)	185 (10.2)	157 (8.6)	173 (9.6)	157 (8.7)	0.28
Mechanical thrombectomy, no. (%)	25 (0.3)	9 (0.5)	7 (0.4)	8 (0.4)	1 (0.1)	0.101
Total cholesterol, mmol/L; median (IQR)	3.96(3.29, 4.72)	3.78(3.14, 4.58)	3.68(3.09, 4.40)	3.94(3.30, 4.58)	4.44 (3.76, 5.17)	<0.001
Triglyceride, mmol/L; median (IQR)	1.37(1.03, 1.89)	0.96(0.77, 1.24)	1.12(0.96, 1.33)	1.47(1.28, 1.72)	2.32(1.92, 2.95)	<0.001
HDL-C, mmol/L; median (IQR)	0.93(0.77, 1.11)	1.04(0.85, 1.25)	0.97(0.81, 1.15)	0.92(0.78, 1.06)	0.82(0.69, 0.96)	<0.001
LDL-C, mmol/L; median (IQR)	2.31(1.72, 2.98)	2.48(1.89, 3.23)	2.22(1.64, 2.89)	2.27(1.70, 2.91)	2.26(1.67, 2.91)	<0.001

IQR denotes interquartile range; SD stands for standard deviation. NAFLD, non-alcoholic fatty liver disease; CHD, coronary heart disease; PAD, peripheral arterial disease; HDL-C, high-density lipoprotein cholesterol; LDL-C, low-density lipoprotein cholesterol.

According to the FLI, 1,350 (18.7%) patients were classified as having possible NAFLD and 3,780 (52.3%) were diagnosed with NAFLD. The crude prevalence of NAFLD among the acute ischemic stroke (AIS) patients is shown in Table 1.

### 3.2 Association between the RC and 3-month poor functional outcome

Among the 7,234 patients, 98.9% completed the 3-month follow-up or were deceased, while 1.1% were lost to follow-up. In the univariate analysis, the rates of the poor functional outcomes (mRS 3–6) at 3 months were 12.1 and 18.5% for the patients in the highest quartile of the remnant cholesterol (RC) levels (Q4), compared to those in the lowest quartile of the RC levels (Q1). The odds ratio (OR) for the poor functional outcomes was 0.98(95% CI: 0.96–1) for the highest quartile (Q4) compared to the lowest quartile (Q1). A significant trend was observed across the RC quartiles and poor functional outcomes (*p* for trend <0.001). This association persisted after adjusting for age, sex, smoking status, alcohol consumption, BMI, hypertension, diabetes, coronary heart disease (CHD), NIHSS score at admission, TOAST classification, antihypertensive medication, and antiplatelet therapy (*p* for trend = 0.039). Details are provided in Table 2.

**Table 2:** Adjusted ORs of the outcomes at 3 months according to RC quartile categories.

mRS score 3–6	Quartiles of remnant cholesterol				P for trend
	Q1	Q2	Q3	Q4	
n (%)	334 (18.5)	293 (16.1)	238 (13.2)	218 (12.1)	
Unadjusted	reference	0.98(0.95–1.00)	0.95(0.93–0.97)	0.94(0.92–0.96)	<0.001
Adjusted	reference	0.99(0.97–1.02)	0.98(0.96–1.00)	0.98(0.96–1.00)	0.039

### 3.3 Subgroup analysis

**Figure 2:**
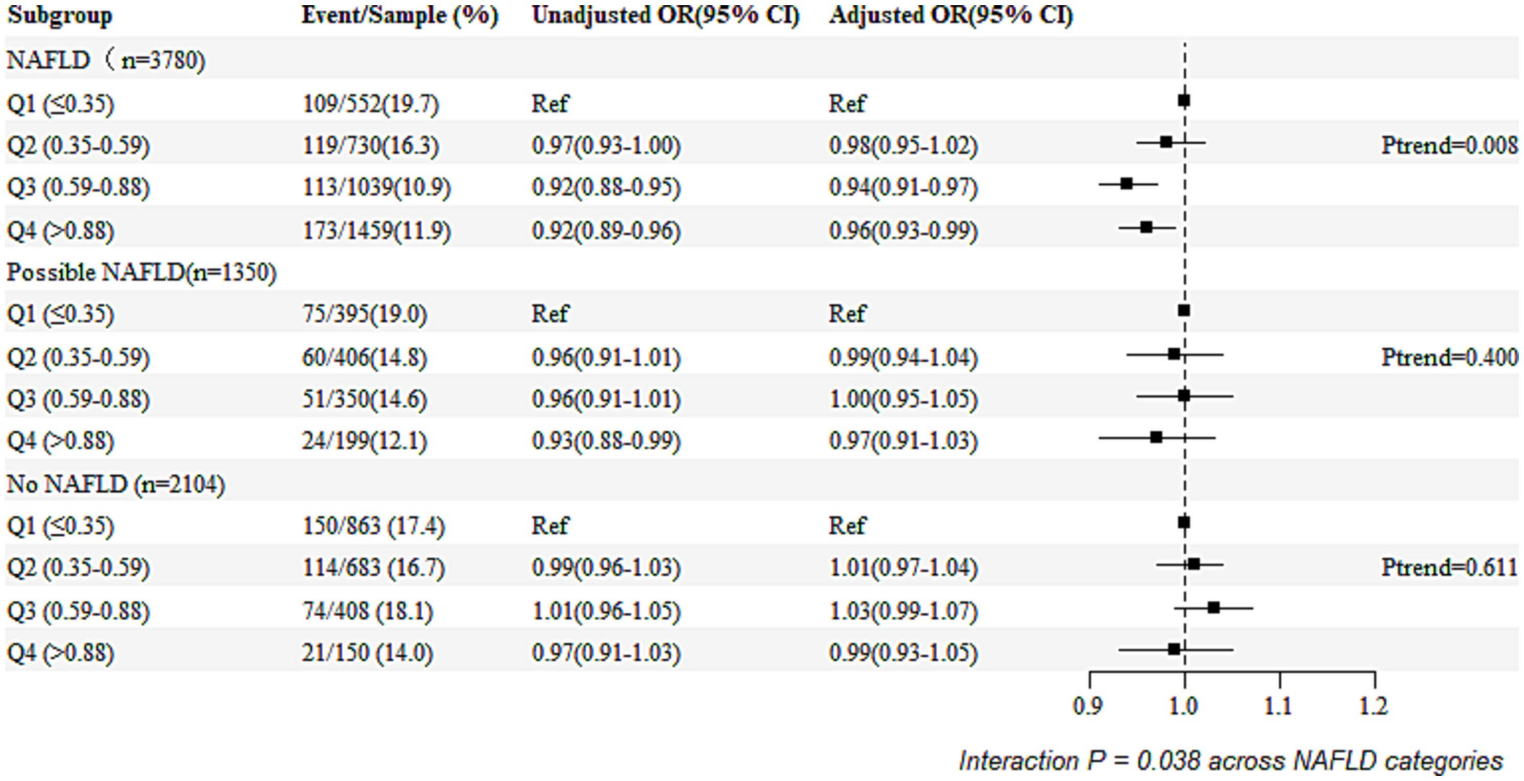
Relationship between NAFLD and poor functional outcomes based on RC quartile categories.

Figure 2 presents the unadjusted and adjusted odds ratios (ORs) and forest plots from the logistic regression analyses examining the association between the remnant cholesterol (RC) percentile groupings (Q1 as the reference group) and the risk of the poor functional outcomes, stratified by NAFLD status. Compared to the individuals in Q1, those in Q2, Q3, and Q4 of the RC had adjusted ORs of 0.98 (95% CI: 0.95–1.02), 0.94 (95% CI: 0.91–0.97), and 0.96 (95% CI: 0.93–0.99), respectively. The ORs were lower in the individuals with NAFLD compared to those without NAFLD or with possible NAFLD (*P* for interaction = 0.038).

### 3.4 Association between the remnant cholesterol and poor functional outcomes

**Figure 3:**
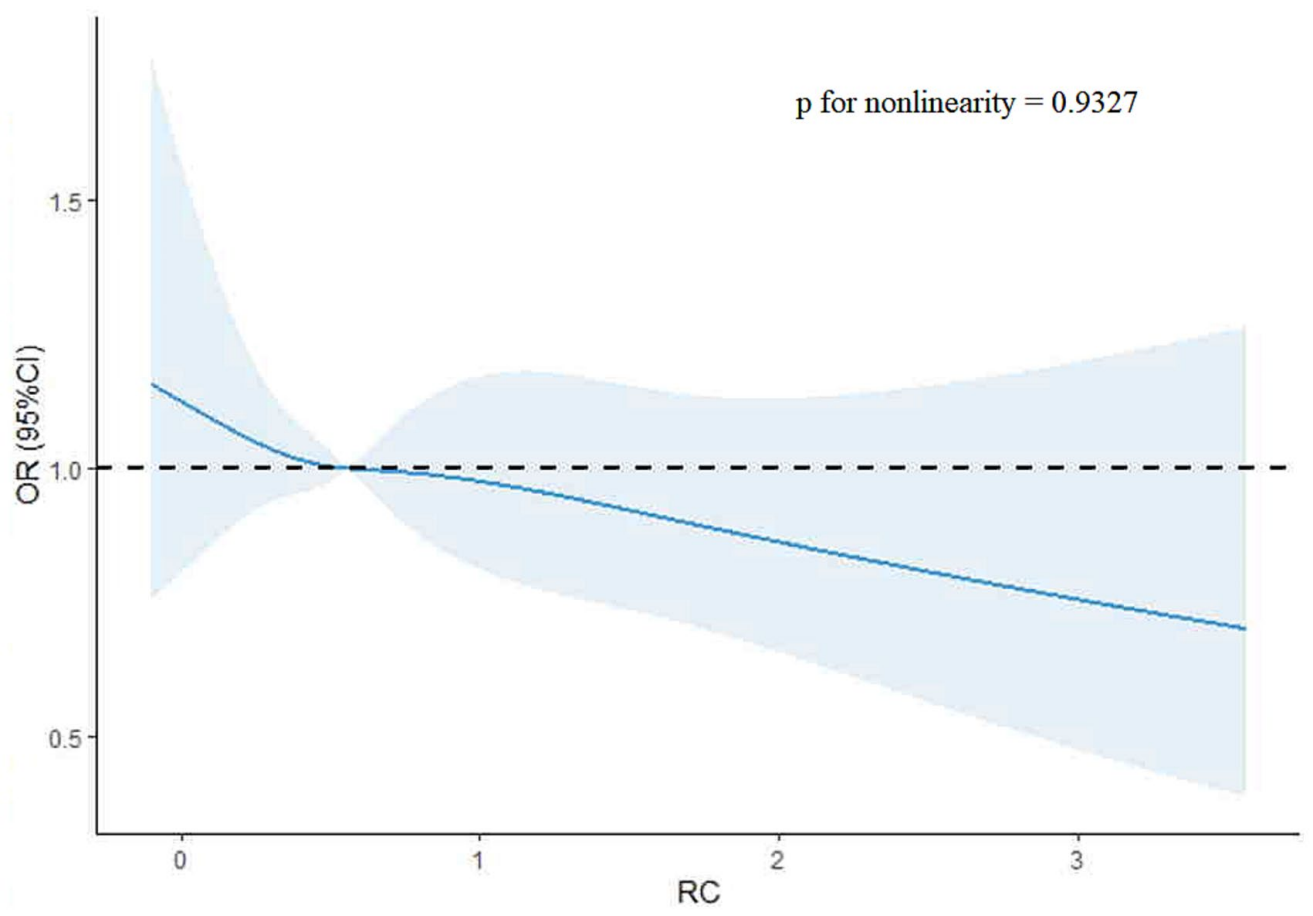
Restricted cubic spline plot of remnant cholesterol and poor functional outcomes.

The restricted cubic spline (RCS) analysis showed no evidence of a non-linear relationship between remnant cholesterol (RC) levels and poor functional outcomes (*p*-value for non-linearity = 0.9327). A linear trend was observed, with an OR of 1 at an RC value of 0.56. The RCS plot is shown in Figure 3.

## 4 Discussion

In this prospective registry study, we found that higher levels of the remnant cholesterol (RC) were associated with a reduced likelihood of the poor functional outcomes in the patients with ischemic stroke. Specifically, a linear relationship between the RC and poor outcomes was observed, with an RC value of 0.56 serving as the threshold where the odds ratio crossed 1. In addition, non-alcoholic fatty liver disease (NAFLD) was found to modify the relationship between the RC and poor functional outcomes, as indicated by a significant interaction (*p*-value for interaction = 0.038). These findings suggest that RC may serve as a potential predictor of functional recovery in ischemic stroke patients, with NAFLD playing a role in modifying this association.

To date, evidence on the relationship between blood lipids and stroke prognosis is limited, with existing results being controversial. A recent retrospective study involving 165 patients who underwent mechanical thrombectomy found that elevated remnant cholesterol was associated with a less favorable prognosis (mRS ≤ 2) in patients with non-large artery atherosclerosis strokes (adjusted OR = 0.31, 95% CI: 0.11–0.85, *p* = 0.023) (23). Conversely, LDL-C has been reported to exhibit a U-shaped relationship with infarct volume in strokes caused by large artery occlusion, with higher early LDL-C concentrations being associated with better clinical outcomes (mRS 0–2) at 3 months (24). Similarly, cholesterol levels were found to be negatively associated with poor outcomes at 3 months (*β* = −0.17, *p* = 0.031) (25). However, these studies were small-sample observational studies and lacked evidence from larger, multicenter studies. The results of the current study are inconsistent with previous findings but align with Hutanu’s research. Interestingly, in our multicenter, nationwide, prospective registry cohort, high levels of the remnant cholesterol were associated with a decreased risk of poor functional outcomes at the 3-month follow-up in patients with NAFLD. However, this relationship was not observed in the stroke patients without NAFLD.

Potential mechanisms may explain why elevated RC levels in the presence of NAFLD could be associated with better stroke outcomes. One possibility is the “obesity paradox,” where a higher body mass index (BMI) is sometimes linked to better outcomes after ischemic stroke (26–28). Our study found that the patients with higher RC levels had higher BMI values at admission, and NAFLD is associated with increased obesity (29). This could suggest that obese patients, due to chronic low-grade inflammation, might develop tolerance to inflammatory responses and hypercatabolic states, which may confer some protection against stroke (30). In addition, cholesterol might serve as a buffer against free radicals released during ischemic injury, potentially limiting infarct expansion (31). Furthermore, brain-derived neurotrophic factor (BDNF), which is critical for the central nervous system’s development and recovery, has been associated with blood lipid levels. Higher BDNF levels have been reported to be correlated with lower HDL and higher triglyceride concentrations (21), which might influence stroke recovery and outcomes.

To the best of our knowledge, this study is the first to explore the association between residual cholesterol and poor prognosis following acute ischemic stroke and its interaction with NAFLD. However, our study has several limitations. First, NAFLD was defined using the fatty liver index because liver biopsy and ultrasonography are not suitable for large sample sizes. Nevertheless, the fatty liver index is a well-established, non-invasive biomarker for predicting hepatic steatosis and has been validated in both Chinese (32) and global populations (20). In addition, as a cohort study, we were unable to explore the detailed pathophysiological mechanisms underlying the effects of RC abnormalities on stroke outcomes in the NAFLD patients. Further research with specifically designed studies is needed to validate and gain a better understanding of these findings. Baseline differences between the included and excluded patients were also observed, but we believe that these differences did not likely substantially affect the results, given our adjustments for potential confounders. However, residual confounding and limited generalizability must be considered when interpreting the findings.

## 5 Conclusion

We found an inverse relationship between RC levels and poor functional outcomes in patients with ischemic stroke, which was influenced by NAFLD. Future studies on optimal target RC levels in patients with NAFLD are needed.

## Data Availability

The raw data supporting the conclusions of this article will be made available by the authors, without undue reservation.
