# Supplementary material for: Impact of remnant cholesterol on acute ischemic stroke prognosis: a nationwide cohort analysis stratified by non-alcoholic fatty liver disease status
Source: Front Neurol. 2025 Jan 30;16:1472871. doi: 10.3389/fneur.2025.1472871 (PMC11822771; doi:10.3389/fneur.2025.1472871)
Supplement: Supplementary file 1 [file Table_1.DOCX]

Supplementary table 1. Comparison of baseline data for included and excluded patients

|  | ***Overall*** | **Included patients** | **Excluded patients** | ***P value*** |
| --- | --- | --- | --- | --- |
| n | 15166 | 7,234 | 7,932 |  |
| AGE (mean (SD)) | 62.23 (11.30) | 61.57 (11.13) | 62.96 (11.44) | <0.001 |
| Male no. (%) | 10364 (68.3) | 5792 (73.0) | 4572 (63.2) | <0.001 |
| BMI (kg/m^2^); mean (SD) | 24.71 (3.34) | 24.73 (3.34) | 24.68 (3.34) | 0.375 |
| Hypertension | 9494 (62.6) | 4890 (61.6) | 4604 (63.6) | 0.012 |
| Diabetes | 3510 (23.1) | 1725 (21.7) | 1785 (24.7) | <0.001 |
| CHD | 1608 (10.6) | 823 (10.4) | 785 (10.9) | 0.355 |
| PAD | 118 ( 0.8) | 63 ( 0.8) | 55 ( 0.8) | 0.885 |
| Renal insufficiency | 131 ( 0.9) | 68 ( 0.9) | 63 ( 0.9) | 0.998 |
| NAFLD no. (%) |  |  |  | 0.019 |
| NAFLD | 4946 (52.8) | 1166 (54.8) | 3780 (52.3) |  |
| Possible NAFLD | 1693 (18.1) | 343 (16.1) | 343 (16.1) |  |
| No NAFLD | 2722 (29.1) | 618 (29.1) | 2104 (29.1) |  |
| Medication, no. (%) |  |  |  |  |
| Antihypertensive | 8053 (53.5) | 4240 (53.8) | 3813 (53.2) | 0.424 |
| Antiplatelet | 440 ( 2.9) | 205 ( 2.6) | 235 ( 3.3) | 0.016 |
| Lipid lowering | 547 ( 3.6) | 321 ( 4.1) | 321 ( 4.1) | 0.003 |
| Total cholesterol, mmol/L, median (IQR) | 3.97 [3.31, 4.72] | 3.98 [3.36, 4.71] | 3.96 [3.29, 4.72] | 0.179 |
| Triglyceride, mmol/L; median (IQR) | 1.37 [1.03, 1.87] | 1.37 [1.03, 1.85] | 1.37 [1.03, 1.89] | 0.603 |
| HDL-C, mmol/L; median (IQR) | 0.93 [0.78, 1.12] | 0.94 [0.78, 1.13] | 0.93 [0.77, 1.11] | 0.019 |
| LDL-C, mmol/L; median (IQR) | 2.31 [1.73, 2.97] | 2.31 [1.75, 2.97] | 2.31 [1.72, 2.98] | 0.939 |

IQR, denotes interquartile range；SD, means standard deviation.

NAFLD, non-alcoholic fatty liver disease; CHD, coronary heart disease; PAD, peripheral arterial disease; HDL-C, high density lipoprotein cholesterol; LDL-C, low density lipoprotein cholesterol; RC, remnant cholesterol.
